# Targeting BTK Signaling in the Microenvironment of Solid Tumors as a Feasible Cancer Therapy Option

**DOI:** 10.3390/cancers13092198

**Published:** 2021-05-03

**Authors:** Justin K. Messex, Geou-Yarh Liou

**Affiliations:** 1 Center for Cancer Research and Therapeutic Development, Clark Atlanta University, Atlanta, GA 30314, USA; jmessex@cau.edu; 2 Department of Biological Sciences, Clark Atlanta University, Atlanta, GA 30314, USA

**Keywords:** Bruton’s tyrosine kinase, cell signaling, tumor microenvironment, cancer therapy, solid tumor

## Abstract

**Simple Summary:**

Protein tyrosine kinase BTK is essential for B cell maturation and proliferation. Dysregulation of BTK signaling in B cells leads to B cell lymphoma. In addition to B cells, BTK is also expressed in other types of immune cells including MDSC, dendritic cells, mast cells and macrophages, all of which comprise the tumor microenvironment in solid cancers. Although BTK inhibitors have been FDA-approved as the front-line treatment for B cell malignancy CLL/SLL, studies have been reluctant to report on BTKs role within the tumor microenvironment during solid cancer development limiting the possibility of using these BTK inhibitors as an adjuvant treatment option for solid cancers. Here, we review BTK signaling within the cells found in the tumor microenvironment as well as summarizing clinical trials using BTK inhibitors which target the tumor microenvironment in an attempt to combat solid tumors.

**Abstract:**

The cell environment plays a pivotal role in determining cellular outcome, as well as cancer initiation, progression, and dissemination. Within this environment, in addition to the structural components, such as the extracellular matrix, there are various types of cells surrounding the tumor cells. Communication among these cells and the tumor cells via signaling pathways is important for tumor growth. Originally discovered in patients with immunodeficiency X-linked gammaglobulinemia, the Bruton’s tyrosine kinase (BTK) signaling pathway, known for its role in B cell maturation, is critical to cancer cell proliferation, metastasis and evasion of cancer eliminating cells. Given that BTK inhibitors have been FDA approved for chronic lymphocytic leukemia/small lymphocytic lymphoma and that the majority of BTK studies have been focused on B cells, the use of BTK inhibitors as a future treatment strategy of solid tumors has yet to be evaluated. In this review, we summarize studies analyzing BTK signaling within the cells found in the tumor microenvironment, as well as clinical trial where BTK inhibitors are currently being used to target the tumor microenvironment as a way to combat solid tumors.

## 1. Introduction

As tumor cells begin to thrive, the microenvironment which they reside in becomes increasingly important to their survival and spread [1,2,3]. The microenvironment composed of immune cells, vessels for transportation, structural components, e.g., the extracellular matrix, signaling proteins, and nerve cells provide necessary resources for tumor proliferation. Although the components of the microenvironment may differ slightly depending on the location of the tumor, each microenvironment plays a role in tumor development by utilizing various signaling pathways to control gene expression.

Under times of stress, the microenvironment undergoes various degrees of alterations in an attempt to maintain conditions that are conducive for tumor growth. Modulation of the tumor microenvironment is orchestrated by a vast network of cell-to-cell signaling. For example, activation of the phosphatase and tensin (PTEN) pathway in CD8^+^ T has been highly correlated with a significant decrease in T cell differentiation and enhancing immune suppression in breast cancer patients [4]. 

B lymphocytes, also known as B cells, originate in the bone marrow. Here, they depend on the signal from a protein tyrosine kinase known as Burton’s tyrosine kinase (BTK) for their maturation [5]. Once mature, they become a part of the adaptive immune response and are responsible for the secretion of antibodies. However, aberrant BTK signaling has been recognized in diseases, like X-linked agammaglobulinemia (XLA), as well as various B cell lymphomas [6]. In this review, we summarize the latest findings of the BTK signaling in the tumor microenvironment of the solid tumors and its potential use for treating solid tumors. 

## 2. Cells of the Tumor Microenvironment

At its core, the tumor microenvironment is supported by the extracellular matrix (ECM). This collage of extracellular molecules, glycoproteins, and collagen provides rigidity to support the tumor cells, as well as remodeling itself to allow expansion of adipose tissue, which is known for its role in colorectal cancer [7,8]. Several factors, including TNF, IL-6, leptin, adiponectin, and visfatin secreted by unregulated adipose tissues and adipocytes, have been shown to enhance cell proliferation, survival, migration, anti-apoptosis and angiogenesis of colorectal cancer [9,10]. Furthermore, the ECM allows multipotent cells like mesenchymal stromal cells (MSCs) to receive signals which allow differentiation into pro-tumor components of the tumor microenvironment [11]. The presence of cytokines, like tumor growth factor β1 (TGFβ1), has also been shown to induce MSC differentiation into carcinoma-associated fibroblasts (CAF), producing a significant increase in the migratory and invasive properties of prostate cancer cells [11].

Mast cells, another type of cell found in abundance within the tumor microenvironment, contributes to tumor development by causing inflammation at the tumor site, allowing nutrients and blood flow to reach the tumor. Studies have shown that protein kinase D2/3 (PKD 2/3) of the prostate cancer cells increased infiltrating mast cells through turning on transcription factors, including AP-1 and/or nuclear factor kappa light chain enhancer of activated B cell (NF-κB), to upregulate scf, CCL5, and CCL11 transcripts [12]. Furthermore, blockade of mast cell recruitment by prostate cancer cells though PKD inhibitors diminished tumor volume and growth in an allografted mouse model. Other cells of the tumor microenvironment include cells of the immune system; however, within the tumor microenvironment, these cells are often exploited by tumorous cells and prevented from doing their job.

In lung cancer, for example, CD3^+^ T cells represent the majority of infiltrating immune cells contributing to tumor progression. Furthermore, CD4^+^ helper T cells and CD8^+^ cytotoxic T cells present in lung cancer contribute to the production of immune exhaustion proteins, including cytotoxic T-lymphocyte antigen 4 (CTLA-4), lymphocyte activated gene 3 (LAG3), programmed cell death protein 1 (PD-1), etc., leading to tolerance of tumor antigens and loss of anti-cancer effects in these cells [13]. Increased levels of IL-17, which contribute to an anti-cancer immune response, as well as CTLA-4 positive regulatory T cells, have also been highly associated with the bronchoalveolar lavage of the cancerous lung, suggesting that the anti-immune response was enhanced in the local cancerous microenvironment advocating for lung cancer progression and metastasis [14]. Other studies using Kras^G12D^;p53^fl/fl^ transgenic mice to study the microenvironment of lung cancer, showed a collection of immune cells infiltrating the cancerous area, including B cells, CD4^+^ and CD8^+^ T lymphocytes, macrophages, CD11c^+^ dendritic cells, and Gr1^+^ neutrophils. Depletion of Gr1^+^ neutrophils using the anti-Gr1 neutralizing antibody resulted in an increase of macrophages, as well as CD4^+^ and CD8^+^ T lymphocytes, with a simultaneous decrease in regulatory T cells. In addition, Gr1^+^ depletion also diminished lung cancer growth and re-sensitized lung cancer cells to anti-PD1 treatment allowing activation and proper function of T cells [15]. Other immune cells of the innate response, such as macrophages, are critical for a healthy immune system. However, they are also known to contribute to the tumor microenvironment in a variety of ways and are widely recognized for the vital role they play in tumor progression [16]. 

**Figure 1:**
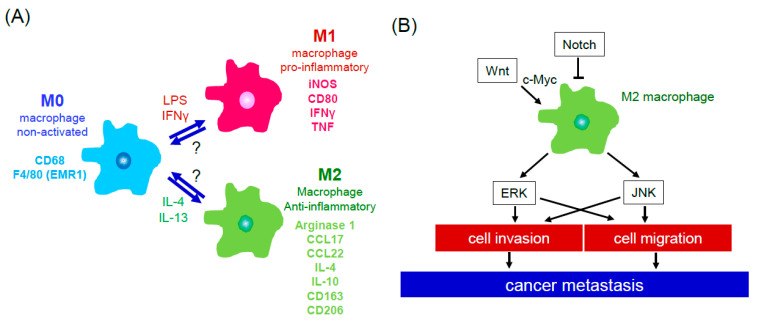
Macrophage polarization and M2 macrophage-associated signaling pathways that contribute to cancer dissemination. (**A**) Non-activated macrophages (M0 macrophages) are polarized to pro-inflammatory/M1 macrophages which specifically express several molecules, including iNOS, CD80, IFNγ, and TNF, in the presence of LPS and IFNγ. In the presence of IL-4 or IL-13, M0 macrophages are polarized to anti-inflammatory/M2 macrophages which specifically express arginase 1, CCL17, CCL22, IL-4, IL-10, CD163, and CD206, and contributing to tissue repair process and tumor development. (**B**) M2 macrophages potentiate cancer metastasis through activation of ERK and JNK signaling that upregulates cell migration and invasion. Notch signaling inhibits M2 macrophage polarization. Wnt signaling can polarize macrophages to a M2 subtype by activation of c-Myc. Abbreviation: ERK: extracellular signal-regulated kinase; JNK: c-Jun N-terminal kinase. Abbreviation: EMR1: EGF-like module-containing mucin-like hormone receptor-like 1; iNOS: inducible nitric oxide synthase; IFNγ: interferon gamma; TNF: tumor necrosis factor; LPS: lipopolysaccharide; IL-4: interleukin-4; IL-10: interleukin-10.

Macrophages in their immature non-activated form, indicated as M0, have two major destinations. Upon stimulation by lipopolysaccharide (LPS) or interferon gamma (IFNγ), M0 macrophages polarize into M1 pro-inflammatory macrophages. On the other hand, M0 macrophages, when exposed to IL-4 or IL-13 cytokines, differentiate into M2 immunosuppressive macrophages and have been known for their role in tumor progression (Figure 1A). Tumor associated macrophages (TAMs) possess a M2-like phenotype and are abundant within the tumor microenvironment of various cancers, like colitis-associated carcinoma, contributing to tumor progression [17,18,19]. 

Mice treated with Azoxymethane (AOM)/Dextran Sodium Sulfate (DSS), used to induce inflammatory colorectal cancer, well recapitulate human colitis-associated cancer of the colon. Blockage of the EGFR signaling pathway present in cancerous cells has been shown to assist in M2 macrophage polarization, reduced tumor growth and showed a reduction in the amount of infiltrating CD206^+^ M2 macrophages in AOM/DSS-induced murine colon cancer mice [20]. This study also showed that the conditioned media of colon cancer cells promoted macrophage polarization to a M2 subtype through an increase in arginase-1, CCL17, CCL22, IL-4, and IL-10. Furthermore, knockout of the EGFR pathway in the colon cancer cells reversed macrophage polarization back to a M1 subtype, suggesting that EGFR signaling of colon cancer cells modulates macrophage polarization and repolarization to promote colon cancer development and progression [20]. In other types of cancers, such as hepatocellular carcinoma (HCC), TAMs express either CD86^+^ (M1 subtype) or CD206^+^ (M2 subtype). However, using this as a diagnostic tool for HCC is uncertain as one study evaluated 253 patients with HCC and discovered the presence of macrophages was not associated with diagnosis nor prognosis of HCC in clinic. They did, however, determine that increased numbers of CD206^+^ TAMs were linked to tumor aggressiveness, defined by the total tumor numbers, tumor-node-metastasis, poor overall survival, and recurrence, contradicting the results found in breast cancer studies [21].

## 3. Cell Signaling in Tumor Microenvironment

Under physiological conditions, healthy cells receive a signaling molecule at the cell surface often leading to the activation of transcription factors, ultimately transcribing genes that function to maintain homeostasis within the cell. However, signaling pathways are on occasion disrupted and altered with pernicious effects. Aberrant signaling may be caused by a number of factors, such as structural changes within the binding sites of a signaling molecule, altered affinity in binding sites of a receptor protein, or any other change that causes the signal to be unregulated. Once altered, signaling pathways initiate a cascade of events that lead to uncontrolled cell proliferation, and the tumor microenvironment provides a safe haven for the growing tumor.

Although signaling within the tumor microenvironment is complex and nuanced, activation of a signaling pathway by a stimulus is largely an attempt to create an area conducive for tumor growth as an end result. For example, the Notch signaling pathway has been indicated as an important macrophage differentiator contributing to the M1/M2 TAM population [22] (Figure 1B). As previously mentioned, M2 macrophages are widely known as sponsors of tumor growth and metastasis through the production of cytokines. Studies have shown that cytokines IL-6 and IL-8 secreted from M2 macrophages produced via the MAPK pathway result in enhanced migratory and invasive abilities of colorectal cancer cells [23]. Conversely, blockade of JNK and ERK signaling pathways in M2 macrophages diminished invasive and metastatic capacities via severe reduction of IL-6 and IL-8 [23]. Furthermore, activation of canonical Wnt/β-catenin signaling was also detected in monocyte to macrophage differentiation, as well as in M2 polarized macrophages. Blockade of the β-catenin pathway in M2 macrophages impeded growth and cell migration of HCC in a co-culture setting, while activation of Wnt signaling in M2 macrophages enhanced polarization through activation of c-Myc. Meanwhile, inhibition of Wnt ligands released from HCC tumor cells suppressed the co-cultured macrophages from being polarized to the M2 subtype, which attenuates tumor growth and progression [24].

Tumor cells also have the ability to recruit neural progenitor cells, pushing their differentiation into adrenergic infiltrating nerves. These newly formed sympathetic nerves release noradrenaline, consequently, promoting angiogenesis using the VEGF signaling pathway while simultaneously promoting expansion of cancer stem cells by exploiting the Wnt/β-catenin pathway [25]. An increase in VEGF expression by M2 macrophages has also been exploited in the FAK/Akt/NF-κB signaling pathways leading to angiogenesis in prostate cancer, as well [16]. 

Other signaling pathways, such as the TGF-β pathway, are thought to diminish anti-tumor responses of CD8^+^ cytotoxic T cell and NK cell; however, the mechanism by which it does so remains unclear. Studies have shown that blockade of the TGF-β pathway in T cells enhanced T cell responses towards tumor antigens, as well as inhibiting tumor development [26]. 

## 4. BTK Signaling

**Figure 2:**
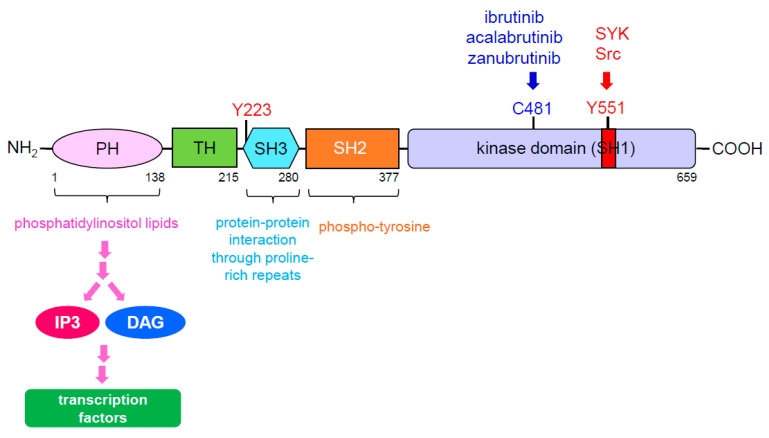
BTK domain diagram. BTK contains five domains, including a PH domain at the amino terminus, a TH domain, a SH3 domain, a SH2 domain and a tyrosine kinase domain at the carboxyl terminus. The PH domain of BTK is able to interact with phosphatidylinositol lipids which allows it to eventually regulate transcription factors through IP3 and DAG. The SH3 domain of BTK mediates protein-protein interactions through proline-rich repeats and also contains a tyrosine at 223 position (Y223), an autophosphoryaltion site of BTK. The SH2 domain of BTK can interact with phospho-tyrosine. The kinase domain of BTK, also known as the SH1 domain contains a tyrosine at 551 position (Y551) with the activation loop. Once Y551 is phosphorylated by SYK or Src, it results in autophosphrylation of Y223 to fully activate BTK. In addition, cysteine 481 (C481) is the site to which BTK inhibitors covalently bound. Abbreviation: PH: pleckstrin homology; TH: Tec homology; SH: Src homology; IP3: inositol triphosphate; DAG: diacylglycerol.

BTK is a member of the Tec family kinases, a family of kinases highly associated with regulation of immune functions. However, unlike Tec kinases, which control T helper cell function, BTK is primarily found in B cells and essential for B cell maturation, as well as being known for various other functions when expressed in other types of cells, such as macrophage polarization [27]. Located on the cytoplasmic side of the B cell, the BTK structure contains five interaction domains, including a pleckstrin homology (PH) domain and a kinase domain with enzymatic activity (Figure 2). Once antigen binds to the B cell receptor, BCR, the PH domain of BTK interacts with phosphatidylinositol triphosphate (PIP3), a downstream signaling activator generated from phosphoinositide 3-kinase (PI3K). Once bound to PIP3, BTK is phosphorylated by the spleen tyrosine kinase (SYK) within the kinase domain, also known as the Src homology type 1 (SH1) domain, at Y551 [28]. BTK then autophosphorylates itself in the SH3 domain at Y223, allowing full activation of BTK [29,30]. In return, two primary secondary messengers, inositol triphosphate (IP3) and diacylglycerol (DAG), are activated, turning on various processes which regulate cellular homeostasis. 

BTK is considered a non-receptor tyrosine kinase and does not receive direct stimulation from antigen. This is why BTK is able to be activated by multiple different antigens. The short cytoplasmic domain of the BCR associates with the disulfide-linked CD79a/CD79b heterodimers upon stimulation from antigen binding [28]. These heterodimers contain immunoreceptor tyrosine based activation motifs (ITAMS) which are phosphorylated upon antigen binding to create docking sites for SYK, a BTK upstream activator as previously mentioned, ultimately leading to the activation of the BTK signaling cascade. In the absence of BCR stimulation and, consequently, the absence of BTK signaling, B cells undergo a high rate of apoptosis due to a reduction in the expression of the anti-apoptotic protein Bcl-xL [31]. However, stimulation of BTK through the BCR antigen binding ultimately leads to the activation of the Akt pathway, subsequently turning on pro-survival transcription factors, including forkhead box O (FOXO) and NF-κB [32]. 

Activation of BTK is also crucial for the function of CXCR4 and CXCR5, G-protein coupled receptors found in B cells which play important roles in B cell entry to lymph nodes [33]. Binding of chemokine to CXCR4 or CXCR5 receptor induces BTK activation, ultimately leading to factors which contribute to B cell homing to the surrounding lymphoid organs. Although these pathways are poorly defined, BTK deficient mice showed significant impairment in adhesion and migration response, suggesting that BTK signaling is critical for proper B cell migration and integrin adhesion [34].

Other receptors, like Toll-like receptors (TLRs), are not only found in B cells but also in myeloid cells, including macrophages, mast cells, etc., and can be utilized for activating the BTK signaling pathway. These receptors recognize conserved regions of bacterial and viral origins, such as the lipopolysaccharide (LPS). Upon TLR binding with antigen, an adaptor protein, like myeloid differentiation primary response (MYD88), interacts with BTK, ultimately activating transcription factors necessary for antibody secretion and immunoglobulin class switching, a critical component to maintaining a healthy immune system [35,36,37,38]. 

**Figure 3:**
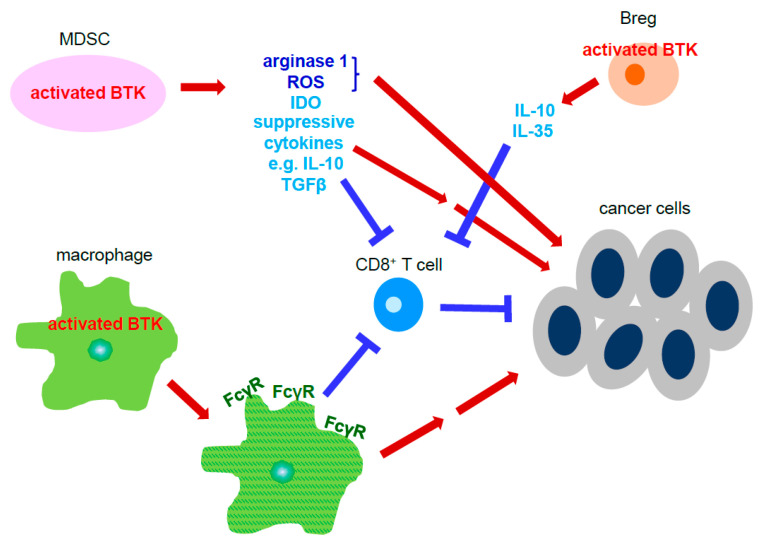
Activated BTK signaling of the stromal cells promotes cancer cell growth and progression. Activated BTK in myeloid-derived suppressor cells increases several molecules, including arginase-1, ROS, IDO, and suppressive cytokines, which directly and indirectly potentiate growth and development of cancer cells. Activated BTK in macrophages induces FcγR expression. FcγR^+^ macrophages can directly promote cancer cell proliferation and metastasis or through inhibition of cytotoxic CD8^+^ T cells, which eliminate cancer cells. Activated BTK in the regulatory B cells is able to elevate cancer cell growth and progression by their secreted suppressive cytokines, including IL-10 and IL-35, that suppress cytotoxic CD8^+^ T cells. Abbreviation: MDSC: myeloid-derived suppressor cell; Breg: regulatory B cells; ROS: reactive oxygen species; IDO: Indoleamine 2, 3-dioxygenase; TGFβ: transforming growth factor β; FcγR: Fcγ receptor.

Although BTK is most well-known for its function in B cell maturation and processes necessary for cellular homeostasis, its contribution to tumor progression by activating cells known for their pro-tumor properties is also well documented (Figure 3). For example, BTK-activated myeloid derived suppressor cells (MDSC) contain immunosuppressive properties which are critical for evading the immune response. The production of arginase-1, indolamine 2, 3-dioxygenase (IDO), nitric oxide (NO), reactive oxygen species (ROS), and suppressive cytokines, such as IL-10 and TGFβ, from MDSCs help tumorous cells avoid death from cells of the adaptive immune response. Studies have shown that IFNγ produced by immune cells to combat harmful material is significantly reduced in the presence of MDSC-generated NO, indirectly promoting tumor growth and progression. Furthermore, activation of BTK was detected in MDSCs isolated from tumor-bearing mice, as well as human samples [39]. 

Activation of BTK has also been detected in the interaction between FcγR-positive macrophages, a type of macrophage with immunosuppressive properties also referred to as M2 macrophages, and B cells in order to promote pancreatic ductal adenocarcinoma (PDAC) growth and progression. Furthermore, activation of BTK in surrounding macrophages was mediated by PI3Kγ protein kinase, resulting in their polarization to FcγR-positive macrophages. Blockade of the signaling pathway using the BTK inhibitor PCI-32765, known as ibrutinib, or PI3Kγ inhibitor TG100-115 decreased PDAC growth both in vitro and in vivo (Table 1). Additionally, combining these two inhibitors with Gemcitabine in the treatment of PDAC tumors halted macrophage infiltration and increased cytotoxic CD8^+^ T cells numbers inside the tumors [40]. Other studies assessing BTK signaling and PDAC development showed that BTK expression in CD1d^hi^CD5^+^ regulatory B cells resulted in the production of IL-10 and IL-35, both of which mediate immunosuppressive functions in regulatory B cells. Inhibition of BTK through its specific inhibitor tirabrutinib diminished both regulatory B cell differentiation and their secreted immunosuppressive cytokines. Furthermore, administration of tirabrutinib to the Kras^G12D^ orthotopic mouse model reduced tumor cell growth and infiltrating regulatory B cells with increasing numbers of CD8^+^IFNγ^+^ T cells, thus also indicating BTK’s role in immune function [41].

Dendritic cells are responsible for bridging the innate and adaptive immune responses, including eradication of cancer, through T cell mediated immunity. Studies using BTK knockout mice demonstrated BTK’s role in regulating dendritic cell development [42]. In addition, it has also been shown that treatment using the BTK inhibitor ibrutinib in murine bone marrow-derived dendritic cells upregulated CD11c in these cells along with MHC-II and CD80 expressions [43]. Stimulation of these ibrutinib-treated dendritic cells with LPS resulted in decreased IL-6, IL-12, and NO production. In addition, these dendritic cells also promoted CD4^+^ T cell proliferation [43]. Altogether, the study suggested that inhibition of BTK in dendritic cells enhanced maturation and activation of dendritic cells and accelerated CD4^+^ T cell growth, which contributes to reshaping the tumor microenvironment during cancer development. 

## 5. BTK Inhibitors and Their Clinic Application for Cancer Therapy

BTK inhibitors, such as ibrutinib, acalabrutinib, and zanubrutinib, are currently being used in clinical trials for hematologic malignancies, including B cell lymphoma, mantle cell lymphoma, and chronic lymphocytic leukemia/small lymphocytic lymphoma, with promising results. Furthermore, the second-generation BTK inhibitor acalabrutinib has also been approved by the FDA for treatment of mantle cell lymphoma, and chronic lymphocytic leukemia/small lymphocytic lymphoma. These inhibitors outcompete ATP by covalently binding to the cysteine 481 residue within the kinase domain to selectively inhibit BTK kinase activity [44,45]. 

Ibrutinib, also known as PCI-32765, was the first BTK inhibitor approved and tested in clinical trials for patients with B cell lymphomas [46,47,48]. In addition to BTK, ibrutinib also shows significant inhibitory activity against 10 other protein kinases, including BLK, BMX, ITK, TEC, EGFR, ERBB2, and JAK 3, with an IC_50_ value less than 100 nM [49]. Furthermore, low dosage of ibrutinib, IC_50_ around10 nM, showed sufficient blockage of BTK autophosphorylation, as well as its downstream signaling targets PLCγ and ERK in cultured B cell lymphoma DOHH2 cells [49]. Several other studies demonstrated that, in human primary chronic lymphocytic leukemia (CLL) cells, ibrutinib is able to not only cause apoptosis through activation of caspase 3 but also block CLL cell proliferation via suppression of BCR- and CD40-activated Akt, ERK, and NF-κB pathways, all of which are downstream targets of BTK [50]. Ibrutinib has also been reported to abolish integrin-mediated adhesion and B cell migration in response to CXCL12 and CXCL13 in CLL cells [50,51,52]. Although ibrutinib has been effective in many cases, CLL patients have encountered drug tolerance with ibrutinib, as well as its off-target effects, resulting in adverse effects like platelet function defect, atrial fibrillation, etc. [53]. 

Acalabrutinib, also known as ACP-196, is a second-generation BTK inhibitor with a higher potency and stronger selectivity than ibrutinib [54,55]. Unlike ibrutinib, acalabrutinib does not inhibit EGFR, ITK or TEC pathways, kinases which closely mimic BTK, and has a lower IC_50_ as compared to ibrutinib [56]. In addition, acalabrutinib shows improved pharmacological features, such as rapid oral absorption and a short half-life, with less adverse effects [56]. In vitro studies have shown that acalabrutinib’s effectiveness against CLL cells is due to inhibiting activation of ERK, NF-κB, and Akt [56]. Prior to clinical trials, acalabrutinib has been evaluated in several animal models of B cell non-Hodgkin lymphoma, including canine model, human NSG primary CLL xenografted mouse model, and TCL1 adoptive transfer mouse model, a mouse model which spontaneously develops CLL-like leukemia and has a similar drug response to human CLL cells [57,58,59,60,61]. Acalabrutinib treated TCL1 transgenic mice showed significant suppression of CLL cell growth and subsequently decreased tumor burden by blocking BCR signaling-mediated PLCγ2 and ERK, proving its in vivo efficacy against CLL [59,60,61]. Furthermore, clinical trials using acalabrutinib for CLL patients indicated acalabrutinib shows less adverse effects in CLL patients than ibrutinib due to its high specificity for BTK and develops resistance far less [59].

As for solid tumors, it has been reported that ibrutinib decreased CD20 mRNA through inhibition of NF-κB transcription factor activity. In addition, ibrutinib also inhibits the expression of decay-accelerating factor (CD55), leading to the complement protein C3d-mediated opsonization of tumor cells [62]. Furthermore, treatment with ibrutinib in breast cancer xenografted mice decreased tumor growth and metastasis [63]. In addition, these treated mice had more mature dendritic cells and less immature MDSCs as compared to the vehicle-treated mice. Using the primary MDSC isolated from mouse, ibrutinib treatment converted primary murine MDSC to dendritic cells as indicated by the various markers of MDSC and dendritic cells. Treatment using ibrutinib also reduced NO generation and cell migration of MDSC. Furthermore, ibrutinib treatment in tumor-bearing mice significantly decreased splenic MDSC and re-sensitizes tumor to anti-PD-L1 immune checkpoint blockade [39]. Although mouse models and in vitro studies have shown high correlation with BTK signaling/inhibition and its effect on tumor progression, clinical studies have been contradictory. In some cases, focusing on BTK inhibition has proven to be a viable therapy for cancer patients while others have shown BTK inhibitors have dismal effects on patient outcome [64].

A phase 2 trial with 77 pancreatic patients, the dual treatment of the BTK inhibitor acalabrutinib and pembrolizumab (PD-L1 inhibitor) was well tolerated and showed a reduction of MDSC cells in the peripheral blood [65]. However, not all trials have shared the same optimistic view for BTK inhibitors. 

Phase 3 clinical trials testing 424 metastatic PDAC patients using ibrutinib plus nab-paclitaxel/gemcitabine as a treatment showed no significant improvement in overall survival nor progression free survival as compared to the current first line treatment, nab-paclitaxel/gemcitabine [66]. Another phase 2 trial with 75 patients for platinum-resistant metastatic urothelial cancer, dual treatment of acalabrutinib and pembrolizumab did not improve the overall survival nor the progressive-free survival of the disease for the platinum-resistant metastatic urothelial cancer as compared to the pembrolizumab treatment alone [67]. The numbers of the peripheral monocytic MDSCs remained same in response to the dual treatment of acalabrutinib and pembrolizumab in comparison to the perbrolizumab alone. Meanwhile, proliferating CD8^+^ T cells increased under the dual treatment. 

Although BTK inhibitors like ibrutinib and acalabrutinib are viable treatment options for lymphoma patients, more pre-clinical studies focusing on BTK inhibition within the TME is necessary to completely understand how it contributes to solid tumor development. This will reveal if these FDA approved BTK inhibitors can serve as an adjuvant treatment for solid tumors.

## 6. Concluding Remarks

Cells found within the tumor microenvironment play a critical role in the initiation, development, and dissemination of cancer. An inextricable web of signaling marries these various types of cells to the tumor microenvironment, while promoting numerous actions that are conducive for tumor growth. In spite of many signaling pathways found in the tumor microenvironment necessary for tumor cell proliferation and survival, the BTK signaling pathway is arguably one of the most vital signaling pathways for cancer progression. The BTK pathway, although necessary for B cell maturation and, as result, vital for proper immune system function, has shown its pertinence as it relates to cancer progression, making it an ideal candidate for exploitation as a cancer therapy. Although some studies analyzing BTK inhibition as a possible cancer therapy have proven inconclusive, our present knowledge of BTK signaling within the tumor microenvironment and its effects on tumor progression, coupled with other studies using BTK inhibitors, prompts us to believe that targeting BTK inhibition within the tumor microenvironment may be a novel therapeutic strategy for patients with solid tumors.

## Figures and Tables

**Table 1:** Summary of BTK inhibitors that have been evaluated in cancer studies.

BTK Inhibitor	Study Type	Cancer Type
ibrutinib	in vitro	B cell lymphoma
		chronic lymphocytic leukemia
		pancreatic cancer
	in vivo	chronic lymphocytic leukemia
		pancreatic cancer
		colorectal cancer
		breast cancer
acalabrutinib	in vitro	chronic lymphocytic leukemia
	in vivo	B cell lymphoma
		chronic lymphocytic leukemia
		pancreatic cancer *
		urothelial cancer *
tirabrutinib	in vivo	pancreatic cancer
zanubrutinib	in vivo	mantle cell lymphoma *

* indicated that the inhibitor is evaluated in the human clinical trials as the in vivo study; P.S. The BTK inhibitors including irbutinib and acalabrutinib are approved by FDA as the frontline treatment only for CLL and possibly other B cell lymphoma.
